# Promising results of an anti-CCR4 antibody, KW-0761, for relapsed Adult T-Cell Leukemia-Lymphoma (ATL)

**DOI:** 10.1186/1742-4690-8-S1-A40

**Published:** 2011-06-06

**Authors:** Atae Utsunomiya, Kensei Tobinai, Kazuhito Yamamoto, Takashi Ishida, Naokuni Uike, Kunihiro Tsukasaki, Kimiharu Uozumi, Masao Tomonaga, Ryuzo Ueda

**Affiliations:** 1 Hematology, Imamura Bun-in Hospital, Kagoshima, Kagoshima, 890-0064, Japan; 2 Hematology and Stem Cell Transplantation, National Cancer Center Hospital, Japan; 3 Hematology and Cell Therapy, Aichi Cancer Center, Japan; 4 Medical Oncology and Immunology, Nagoya City University, Japan; 5 Hematology, National Kyushu Cancer Center, Japan; 6 Hematology and Molecular Medicine, Nagasaki University, Japan; 7 Hematology and Immunology, Kagoshima University, Kagoshima, Kagoshima, 890-0064, Japan; 8 Internal Medicine, Japanese Red Cross Nagasaki Genbaku Hospital, Japan

## Background

ATL is an aggressive T-cell malignancy caused by the virus HTLV-1 with a very poor outcome. ATL, and is characterized by its cell surface expression of CC chemokine receptor 4 (CCR4), to which KW-0761, a defucosylated, humanized antibody with enhanced antibody-dependent cellular cytotoxicity (ADCC), binds. In a phase I study of KW-0761 in 13 patients (pts) with CCR4-positive relapsed ATL, encouraging efficacy of KW-0761 was observed (ORR of 31%; 2CRs and 2PRs, ref. 3). Here, we report the result of a pivotal phase II study of KW-0761 in pts with CCR4-positive relapsed ATL.

## Results

A multicenter phase II study of KW-0761 has been conducted for pts with CCR4 positive, relapsed aggressive ATL with the primary endpoint being overall response rate (ORR). Pts were planned to receive intravenous infusions of KW-0761 at 1.0 mg/kg once a week for 8 weeks. Twenty-eight pts were enrolled, among whom, 27 had at least one infusion of KW-0761. Most frequent adverse events (AEs) were mild to moderate in severity. The most frequent drug-related AEs were lymphopenia, acute infusion reaction, fever, skin rash, chill, thrombocytopenia and neutropenia. Among the 26 pts evaluable for efficacy, the ORR was 50% with 8 CRs and 5 PRs with response rates in each affected lesion being 100% (13/13) for peripheral blood, 63% (5/8) for skin, and 25% (3/12) for lymph node disease, respectively. Updated data including progression-free survival and overall survival, will be presented at the meeting with additional results of prognosis in the phase I study.

